# RNA-Seq reveals differentially expressed genes affecting polyunsaturated fatty acids percentage in the Huangshan Black chicken population

**DOI:** 10.1371/journal.pone.0195132

**Published:** 2018-04-19

**Authors:** Shaohua Yang, Ying Wang, Lulu Wang, Zhaoyuan Shi, Xiaoqian Ou, Dan Wu, Xinmiao Zhang, Hao Hu, Jia Yuan, Wei Wang, Fuhu Cao, Guoqing Liu

**Affiliations:** 1 College of Food Science and Engineering, Hefei University of Technology, Hefei, Anhui, P. R. China; 2 Agricultural Products Quality and Safety Supervision and Management Bureau, Xuancheng, Anhui, P. R. China; Wageningen UR Livestock Research, NETHERLANDS

## Abstract

Fatty acids metabolic products determine meat quality in chickens. Identifying genes associated with fatty acids composition could provide valuable information for the complex genetic networks of genes with underlying variations in fatty acids synthesis. RNA sequencing (RNA-Seq) was conducted to explore the chicken transcriptome from the thigh muscle tissue of 6 Huangshan Black Chickens with 3 extremely high and low phenotypic values for percentage of polyunsaturated fatty acids (PUFAs). In total, we obtained 41,139,108–44,901,729 uniquely mapped reads, which covered 74.15% of the current annotated transcripts including 18964 mRNA transcripts, across all the six thigh muscle tissue samples. Of these, we revealed 274 differentially expressed genes (DEGs) with a highly significant correlation with polyunsaturated fatty acids percentage between the comparison groups based on the ratio of PUFA/SFA. Gene ontology and pathway analysis indicated that the DEGs were enriched in particular biological processes affecting fatty acids metabolism, biosynthesis of unsaturated fatty acids (USFAs), and cell junction-related pathways. Integrated interpretation of differential gene expression and formerly reported quantitative trait loci (QTL) demonstrated that *FADS2*, *DCN*, *FRZB*, *OGN*, *PRKAG3*, *LHFP*, *CHCHD10*, *CYTL1*, *FBLN5*, and *ADGRD1* are the most promising candidate genes affecting polyunsaturated fatty acids percentage.

## Introduction

During recent decades, the breeding of meat type poultry focused on increasing growth performance and improving breast and thigh meat yields. Although the impressive progress made in these meat quality traits, there were some poor performances, such as excessive deposition of abdominal fat, deterioration of taste quality, and decreased sensory acceptability for consumers. As an indigenous breed in China, the Huangshan Black Chicken has a distinct appearance and quality in meat and egg products. Compared with the Arbor Acres (AA) broiler, the Huangshan Black Chicken is highly popular in China because of its polyunsaturated fatty acids concentration. Therefore, the elucidation of the precise molecular mechanisms underlying fatty acids traits in Huangshan Black chickens will have both economic and biological consequences.

In the past several decades, candidate gene analysis, quantitative trait locus (QTL) mapping, and genome-wide association studies (GWASs) have been the main approaches to identify genes or causal mutations for meat quality traits in chickens. A large number of promising genetic associations and genomic regions have been successfully identified. As of December 21, 2017, 8,363 QTLs for 383different traits have been reported in 277 papers in chickens (http://www.animalgenome.org/cgi-bin/QTLdb/GG/index). Of these, 339 QTLs for abdominal fat traits and 144 for breast muscle traits have been detected in the chicken chromosomal regions. Moreover, GWASs can be used to identify the genes and variants underlying important traits more precisely [1–2]. In chickens, GWASs have already been performed for egg production and quality [3], growth [4–5], meat quality [6–7] and disease resistance traits [8–9]. Although these techniques have contributed significantly to our better understanding of mechanisms underlying meat quality traits [6,7], several potential limitations still exist. One major limitation is the fine mapping required to identify causative variants. Additionally, some novel genes or biological pathways associated with the target trait may be excluded unintentionally.

In recent years, next generation sequencing (NGS) technologies are increasingly being used for identifying differential expression as well as opportunities to explore novel transcripts [10]. Of these, RNA-Seq has been widely utilized to detect differentially expressed genes (DEGs) between two gene expression patterns, causative variants, and alternative splicing events. In chickens, many studies of RNA-Seq have been conducted using intestinal mucosal [11], heart [12], uterine [13], and ovarian tissues [14]. However, limited studies on the transcriptome of thigh muscle tissues have been reported. The identification of DEGs in thigh muscle tissue represents the first step toward clarifying the complex biological properties of meat quality traits. Therefore, the regulation of fat deposition in chickens at a genome wide level remains to be elucidated. In the present study, we used RNA-Seq technology to examine the genome-wide transcription profile in thigh muscle tissues between two groups of Huangshan Black Chickens with extremely high and low phenotypic values of polyunsaturated fatty acids. We then proposed key candidate genes affecting polyunsaturated fatty acids percentage by conducting integrated analysis. The identified candidate genes could lead to improved selection of chicken while providing new insights into meat quality traits.

## Materials and methods

### Ethics statement

All procedures for animal handling were reviewed and approved by the Institutional Animal Care and Use Committee (IACUC) of Hefei University of Technology (Permit Number: DK838).

### Animals diet and sample collection

Huangshan Black Chickens were maintained in free-ranging flocks in a standardized farm (Anhui conservation farm for Huangshan Black Chicken, Huangshan, China), using a diet as: maize 64.0%, wheat bran 16.0%, full-fat soybean 10.0%, fish meal 5.0%, feed yeast powder 2.0%, bone meal 1.5%, inorganic additives 0.7%, Lysine 0.3%, Methionine 0.2%, salt 0.3%. Ten male chickens with an average weight of 1.82 kg at 120 days old were selected randomly for our detection. To keep the environment factors identical, all chickens were raised in the same way which was free access to food and water in natural lighting. Performance traits of Huangshan Black chickens were shown in supporting information (S1 File).

12 h after feed was withheld, the chickens were handled by electroshock, bled, and dismembered. The thigh muscle tissue samples from the left leg of the chickens were removed within 30 min after slaughter. The samples for each chicken were carefully collected into an RNase-free tube for RNA isolation, immediately frozen in liquid nitrogen, and kept at −80 °C until required for RNA isolation. Meanwhile, sufficient samples were minced and kept at −20 °C for fatty acids analysis.

### Fatty acids analysis

Fatty acids samples from ten different individuals were methylated according to Ren et al [15] with some modifications. 1 mL of 1 M KOH-methanol was added to the lipids (100 μL) for esterification at 65 °C for 30 min. Then, after being cooled to room temperature, a 2 mL mixture of boron fluoride-methanol (140 g BF_3_-ether per liter of methanol) was added to deal with the fatty acids, and then heated at 65 °C for 5 min. 1 mL of saturated NaCl solution and 1 mL n-hexane were then added at room temperature. The liquid was allowed to separate into 2 phases using a centrifuge (Thermo Scientific, Wilmington, DE, USA) at 2000 rpm for 5 min. The upper phase containing fatty acid methyl esters (FAMEs) was collected.

The FAMEs samples were subsequently analyzed by Gas Chromatography-Mass Spectrometer (GC-MS) using an Agilent 5975C GC (Santa Clara, CA, USA) equipped with a quadruple mass spectrometer (flame ionization detector) and a capillary polar HP-88 cyanopropyl column (60 m × 0.25 mm ID × 0.20 μm film). With a flow rate of 2 mL / min, Helium was used as the carrier gas. Initial column temperature was maintained at 125 °C, 8 °C per minute from 125 °C to 145 °C, then raised to 220 °C at 2 °C / min and maintained for 67 min. Meanwhile, the temperatures of the injector and FID detector were both set at 250 °C. As the internal standard, nonadecanoic acid (C19:0) (Sigma, Saint Louis, MO, USA) was used to quantity the fatty acids. The details of the ten samples were detected as shown in supporting information (S2 File). Of these, six samples (polyunsaturated fatty acid high (FAH): FAH1, FAH2, FAH3; polyunsaturated fatty acid low (FAL): FAL1, FAL2, FAL3) were divided into two groups with extremes of the phenotypic values for PUFA/(SFA+USFA) to detect DEGs for sequential analyses.

### RNA isolation and quality assessment

The thigh muscle tissues of six samples were disrupted with liquid nitrogen and total RNA was extracted with TRIzol reagents (Invitrogen, Carlsbad, CA, USA) following the manufacturer’s instructions [16]. Using the RNase-free DNase I (Invitrogen, Carlsbad, CA, USA), DNA contamination was removed from the RNA by incubating for 30 min at 37 °C. The purity and concentration of the RNA samples were assessed on a NanoPhotometer^®^ spectrophotometer (Thermo Scientific, Wilmington, DE, USA). The integrity of the RNA samples was assessed with the RNA Nano 6000 Assay Kit of the Bioanalyzer 2100 system (Agilent Technologies, CA, USA).

### Library preparation and RNA sequencing

As input material, a total of 3 μg RNA from per sample was used for the RNA sample preparations. The transcriptome library for sequencing was constructed using the NEBNext^®^ Ultra^™^ RNA Library Prep Kit for Illumina^®^ (NEB, USA) according to the manufacturer’s instructions. Using the TruSeq PE Cluster Kit v3-cBot-HS (Illumina) following the manufacturer's recommendations, the index-coded samples were clustered on a cBot Cluster Generation System. After cluster generation, the library preparations were sequenced using an Illumina HiSeq 2000 platform and 100 bp paired-end reads were generated; this was followed by FASTQ file generation and the failed reads elimination by CASAVA ver.1.8.2 (Illumina).

### Quality control for paired-end reads

Using CASAVA ver.1.8.2 (Illumina), the sequencing-derived raw images were transformed into raw reads by base calling. The raw reads were cleaned by our self-written Perl scripts. In this step, low quality reads (more than half of the reads with a phred base quality score of less than 5) were removed to obtain clean reads. In addition, the description statistics for the clean data, such as Q20, Q30, and GC-content were calculated for high-quality downstream analysis. All downstream analyses were based on the clean reads.

### Reads mapping to the reference genome

The chicken reference genome UMD 4.1 and model annotation files were downloaded directly from the website (ftp://ftp.ensembl.org/pub/release-75/fasta/gallus_gallus/dna/) to be utilized for the assembly. The index of the reference genome was built using Bowtie v2.2.3 and paired-end clean reads for each individual chicken were aligned to the reference genome using TopHat v2.0.12. Additionally, HTSeq v0.6.1 was used to count the reads numbers mapped to each gene.

### Identification of DEGs

Differential expression analysis of different groups (the high and low groups with phenotypic values for percentage of polyunsaturated fatty acids) was performed using the DESeq R package (1.10.1). Using a generalized linear model based on the negative binomial distribution, DESeq2 provides statistical counts for determining DEGs in digital gene expression data. Furthermore, the Hochberg and Benjamini method was used to adjust the p-values to control for the false discovery rate. The fold changes (in log 2 scale), p-values, and q-values (corrected p-values) of the DEGs were acquired in the output files from DESeq2. An adjusted p-value of 0.05 was assigned as the threshold for significant differential expression.

### GO and gene functional analysis of DEGs

GO and pathway enrichment analysis of DEGs was implemented in the GOseq R package (version 2.12), in which gene length bias was corrected. GO terms and KEGG pathways (http://www.genome.jp/kegg/) with p-value less than 0.01 were considered significantly enriched among the differential expressed genes.

### Validation of RNA-Seq results with qRT-PCR

To validate the sequencing results, qRT-PCR was carried out on 10 randomly selected DEGs. Using identical samples with RNA-seq, total RNA was converted to cDNA with Superscript III (Invitrogen, CA, USA) following the manufacturer’s instructions and was used as PCR templates. Primers were designed via Primer3 (http://bioinfo.ut.ee/primer3-0.4.0/primer3/input.htm) and are shown in S3 File. qRT-PCR was carried out in triplicate with the LightCycler^®^ 480 SYBR Green I Master Kit (Roche Applied Science, Penzberg, Germany) in a 15 μ L reaction on a LightCycler^®^ 480 (Roche), using the following program: 95 °C for 8 min; 45 cycles of 95 °C for 10 s, 60 °C for 15 s, and 72 °C for 10 s; 72 °C for 10 min. The mRNA levels of the DEGs were normalized by the housekeeping genes GAPDH, β-actin and 18s rRNA, in the corresponding samples. The relative gene expression values were calculated using the 2^−ΔΔCt^ method. Finally, the correlations between RNA-Seq for 10 genes and the mRNA expression level from qRT-PCR were estimated using R (V3.2).

## Results

### Fatty acids profiles determined by GC-MS

A typical chromatogram for the analysis of the 37-component FAMEs reference standard was shown in S4 File. Interestingly, Compared with the AA broiler, the Huangshan Black Chicken displayed higher polyunsaturated fatty acids percentage and intramuscular fat ratio (as shown in S1 File). Meanwhile, all the target compounds can be baseline separated by HP-88GC column with excellent peak shape as indicated in the chromatogram. Meanwhile, the fatty acids profiles of different chicken thigh muscle tissues in the comparison group of FAH and FAL was shown in Table 1. The analysis of the fatty acid profile showed a higher level of PUFAs in the group FAH. Particularly, contents of C22:6n-3 was higher (p<0.01), while that of C18:2n-6, C20:3n-3 and C20:4n-6 were relatively higher (p<0.01) in comparison to the FAL group.

**Table 1:** Analysis of FAMEs in thigh muscle of Huangshan Black chickens in the group FAH and FAL (Mean±SD).

Items	FAH	FAL
C14:0	0.331±0.057^**A**^	0.648±0.119^**B**^
C16:0	19.511±0.686	21.205±0.608
C18:0	19.668±0.785^**a**^	15.944±1.751^**b**^
C20:0	0.305±0.070	0.216±0.057
C14:1	0.088±0.031^**A**^	0.169±0.016^**B**^
C16:1	1.368±0.260^**A**^	4.352±1.331^**B**^
C18:1n-9	22.385±1.425	26.555±1.009
C18:1n-7	2.938±0.507	2.901±0.191
C20:1	0.291±0.042	0.257±0.063
C18:2n-6	18.444±0.199^**a**^	15.542±0.375^**b**^
C18:3n-6	0.150±0.049^**a**^	0.214±0.058^**b**^
C18:3n-3	0.202±0.055	0.265±0.033
C20:2n-6	0.236±0.113	0.238±0.080
C20:3n-3	0.265±0.280^**a**^	0.168±0.131^**b**^
C20:4n-6	9.619±0.086^**a**^	7.727±1.156^**b**^
C20:5n-3	0.115±0.065^**A**^	0.223±0.264^**B**^
C22:6n-3	3.273±0.488^**A**^	2.449±0.193^**B**^
SFA	39.815±1.338	38.012±1.344
PUFA	32.305±0.236	26.827±1.364
PUFA/SFA	0.812±0.030^**a**^	0.672±0.032^**b**^

**Note:** SFA = C14:0 + C16:0 + C18:0 + C20:0; MUFA = C14:1 + C16:1 + C18:1 + C18:1 + C20:1; PUFA = C18:2 + C18:3 + C18:3 + C20:2 + C20:3 + C20:4 + C20:5 + C22:6; USFA = MUFA + PUFA.

Means in the same row with different lowercase superscripts are different at P<0.05; means in the same row with different uppercase superscripts are different at P<0.01.

### RNA sequencing of thigh muscle tissue

We acquired a total of 358.88 million clean reads with an average of 59.81 million (range, 57.31 to 62.63 million) for each sample (Table 2). Alignment of the sequence reads against the chicken reference genome UMD 4.1 yielded 69.01–74.15% of uniquely aligned reads across the six samples, of which 71.3–80.0% fell in annotated exons, 5.2–8.2% were located in introns, and the remaining 14.8–20.5% were assigned to intergenic regions (S5 File). The data sets analyzed are available in the NCBI GenBank (https://www.ncbi.nlm.nih.gov/genbank) and the BioProject ID is PRJNA412788. Additionally, 18,964 mRNA transcripts were detected. Furthermore, the correlation coefficient (R^2^) between the six individuals within the FAH and FAL groups was calculated based on the FRPM value of each sample and was shown to be 0.946 and 0.969, respectively, indicating that the similarity of the three biological samples within each group was sufficiently high (S6 File).

**Table 2:** Basic sequencing data statistics for each sample.

Sample name	FAH1	FAH2	FAH3	FAL1	FAL2	FAL3
Total reads	57317544	59138110	58255332	62638402	61960816	59609516
Total mapped	43427854 (75.77%)	43991875 (74.39%)	43030728 (73.87%)	45961284 (73.38%)	45404156 (73.28%)	42284978 (70.94%)
Multiple mapped	925207 (1.61%)	949905 (1.61%)	979651 (1.68%)	1059555 (1.69%)	1288689 (2.08%)	1145870 (1.92%)
Uniquely mapped	42502647 (74.15%)	43041970 (72.78%)	42051077 (72.18%)	44901729 (71.68%)	44115467 (71.2%)	41139108 (69.01%)
Non-splice reads	26318929 (45.92%)	25224783 (42.65%)	25084799 (43.06%)	26133024 (41.72%)	25612313 (41.34%)	23134101 (38.81%)
Splice reads	16183718 (28.24%)	17817187 (30.13%)	16966278 (29.12%)	18768705 (29.96%)	18503154 (29.86%)	18005007 (30.2%)

### Top genes expressed in the thigh muscle tissue

**Fig 1:**
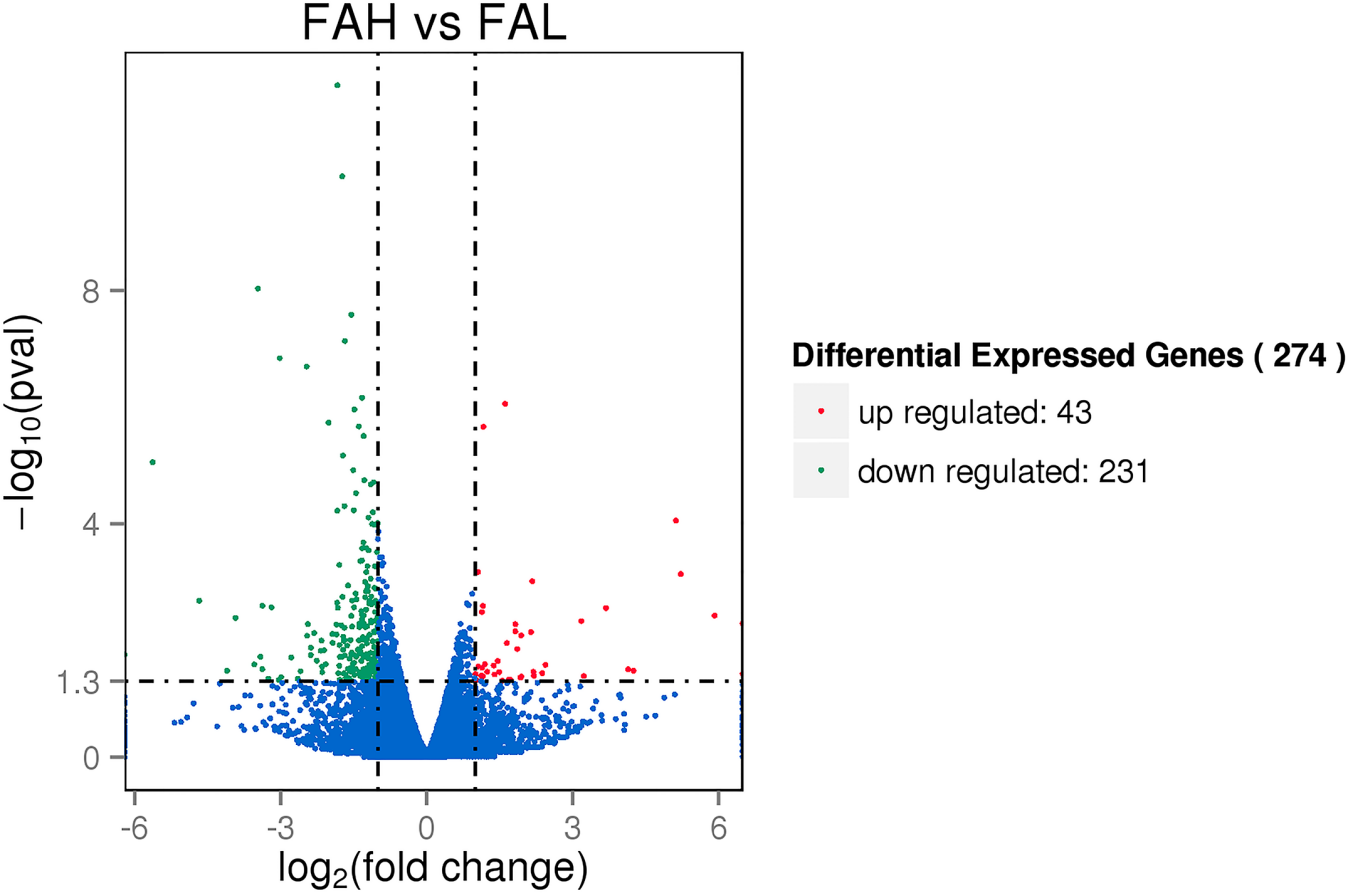
Volcano plot displaying DEGs within two different comparison groups. Note: the y-axis shows the mean expression value of log_10_(q-value), and the x-axis displays the log_2_fold change value. The blue dots represent the transcripts did not reach statistical significance (q > 0.05); the red dots represent whose expression levels were significantly different (q < 0.05).

The differential gene expression profile between FAH and FAL was examined using the RPKM method. In total, 274 DEGs were detected significantly different between the FAH and FAL groups. Of these, 43 genes were up regulated while 231 genes were down regulated. A volcano plot of the two comparison groups that are differentially expressed and illustrate distinct transcriptional profiles is displayed in Fig 1. Moreover, using integrated analysis of RNA-Seq and gene function, the top 20 genes with the highest absolute value of expression in the thigh muscle tissue between FAH and FAL are shown in Table 3. Strikingly, the fat associated genes *FADS2*, *OGN*, and *CD2* accounted for a significant proportion.

**Table 3:** Top 20 expressed genes in thigh muscle tissue with high polyunsaturated fatty acids percentage compared to low polyunsaturated fatty acids percentage.

Symbol	CHR	No. Reads	Log2 fold change	Gene name	q-value	Gene function
FADS2	5	513.11	-1.15	Fatty acid desaturase 2	2.13E-05	Regulated unsaturation of fatty acids, fatty acid beta-oxidation)
DCN	1	2875.33	-1.83	Decorin	5.35E-08	Collagen fibril assembly, tumor suppression
ABI3BP	1	1296.44	-1.68	ABI family member 3 binding protein	7.36E-08	Heparin binding and glycosaminoglycan binding
NAV3	1	258.74	-1.20	Neuron navigator 3	7.83E-05	ATPases associated with a variety of cellular activities
PRKAG3	1	78.24	-1.22	Protein Kinase AMP-Activated Non-Catalytic Subunit Gamma 3	1.33E-05	cell proliferation, cell differentiation in immune responses
OGN	12	1928.36	-1.33	oglycin	6.92E-07	Ectopic bone formation and osteoblast differentiation
CYTL1	4	47.33	-3.02	Cytokine like 1	1.45E-07	Receptor binding bear the CD34 surface marker
CHCHD10	15	9009.06	1.17	Coiled-coil-helix-coiled-coil-helix domain containing 10	2.17E-06	Cristae morphology maintenance, oxidative phosphorylation, and Mitochondrial protein import
FRZB	7	729.84	-1.29	Frizzled-related protein	3.12E-06	Involved in the regulation of bone development
ADGRD1	15	76.24	-1.33	Adhesion G protein-coupled receptor D1	4.27E-04	Transduced extracellular signals through G proteins
FBLN5	5	283.79	-1.12	Fibulin 5	1.01E-04	Promoted adhesion of endothelial cells
CD2	1	47.52	-1.46	CD2 molecule	3.12E-02	Immune recognition with LFA3 on antigen presenting cells
LHFP	1	508.47	-1.08	Lipoma HMGIC fusion partner	1.04E-04	A gene associated with translocation-associated lipoma
BMPER	2	84.83	-1.72	BMP binding endothelial regulator	7.97E-03	Inhibited osteoblast differentiation of the chondrogenic cells
LAMB1	1	2491.65	-1.01	Laminin subunit beta 1	9.75E-05	Cell adhesion, differentiation, signaling and metastasis
FAP	7	445.87	-1.41	Fibroblast activation protein	1.43E-03	Fibroblast growth or epithelial-mesenchymal interactions
LUM	1	1487.66	-1.73	Lumican	9.72E-07	Fibril organization, circumferential growth and tissue repair.
SOD3	4	453.06	-1.29	Superoxide dismutase 3	9.78E-03	Antioxidant enzyme that catalyzed the conversion of superoxide radicals into hydrogen peroxide and oxygen
FSTL1	1	984.73	-1.23	Follistatin like 1	1.12E-03	An autoantigen associated with rheumatoid arthritis
CD38	4	118.45	-1.18	CD38 molecule	4.12E-03	An intracellular calcium ion mobilizing messenger

### Validation of differentially expressed genes

**Fig 2:**
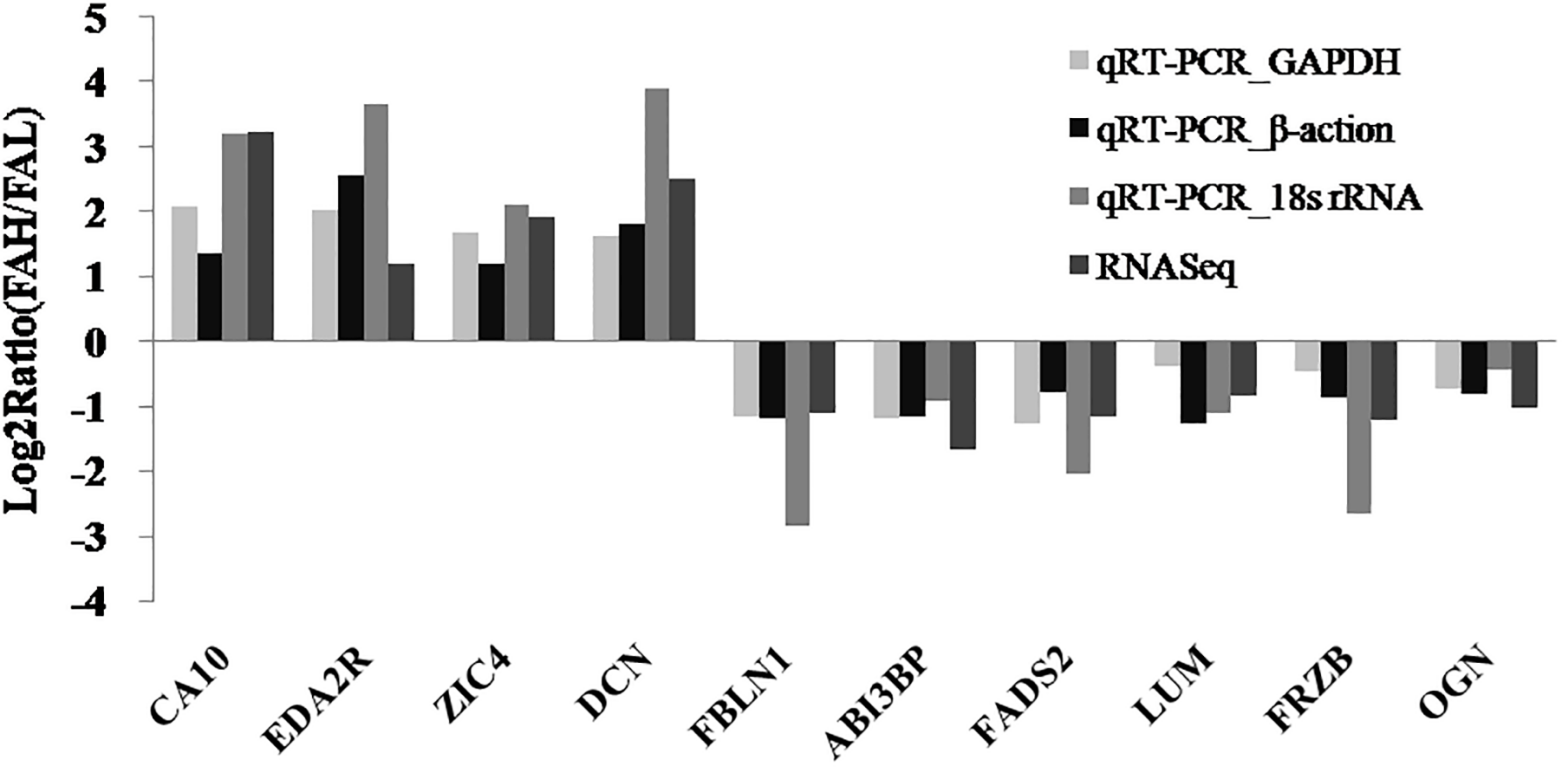
Correlations of mRNA expression level of 10 randomly DEGs between high and low polyunsaturated fatty acids percentage using RNA-Seq and qRT-PCR. Note: the x- and y-axis correspond to the log_2_ (ratio of FAH/FAL) measured by RNA-Seq and qRT-PCR, respectively.

To validate the RNA-Seq results, 10 random DEGs including *FADS2*, *ABI3BP*, *FBLN1*, *DCN*, *LUM*, *FRZB*, *OGN*, *CA10*, *EDA2R*, and *ZIC4* were selected for qRT-PCR analysis. The correlations between the mRNA expression level from qRT-PCR and RNA-Seq were all consistent (Fig 2), validating the reproducibility and repeatability of gene expression data in this study.

### Gene ontology enrichment and pathway analysis

To further study the functional associations of the detected 20 DEGs, gene ontology (GO) analysis was performed. Several important GO categories were enriched (p < 0.01), including GO processes related to synthesis, transport, and metabolic processing of lipids. For fatty acids traits, the important pathways identified were ‘fatty acids metabolic process,’‘acyl-CoA desaturase activity’,‘lipid biosynthetic process,’ and ‘unsaturated fatty acids biosynthetic process,” which also involved several candidate genes. The detailed gene function and pathway analysis of genes are shown in Table 4.

**Table 4:** Summary of the GO analysis of 20 differentially expressed genes.

GO ID	GO term	No. of DEGs	P-value
GO:0031012	extracellular matrix	4	1.09E-22
GO:0006631	fatty acids metabolic process	2	4.18E-18
GO:0016215	acyl-CoA desaturase activity	3	1.27E-06
GO:0022610	biological adhesion	5	1.38E-06
GO:0008610	lipid biosynthetic process	3	3.62E-06
GO:0009888	tissue development	6	1.78E-05
GO:0006636	unsaturated fatty acids biosynthetic process	2	1.56E-04
GO:0010811	positive regulation of cell-substrate adhesion	4	2.08E-04
GO:0061049	cell growth involved in cardiac muscle cell development	3	3.69E-03
GO:0048731	system development	5	2.75E-02
GO:0001676	long-chain fatty acids metabolic process	4	3.21E-02

### Candidate genes

Integrated analysis of DEGs, GO, and pathway results, QTL databases and gene function allows us to suggest *FADS2*, *DCN*, *FRZB*, *OGN*, *PRKAG3*, *LHFP*, *CHCHD10*, *CYTL1*, *FBLN5*, and *ADGRD1* as the 10 promising candidate genes for affecting fatty acids concentration. In addition, 10 genes (*ABI3BP*, *NAV3*, *LUM*, *CD2*, *BMPER*, *LAMB1*, *FAP*, *SOD3*, *FSTL1*, and *CD38*) were also revealed to be associated with fatty acids traits by the significant expression levels of DEGs and the QTL databases. The details of the above candidate genes identified are listed in Table 2.

## Discussion

Polyunsaturated fatty acids play vital roles in multiple physiological processes, they participate in structural functions as major components of biomembranes [17], metabolic energy production, ligands for transcription factors, and messengers in cellular pathways [18]. Additionally, they can regulate the metabolism of lipids and promote the growth and development of animals. For poultry, the content of polyunsaturated fatty acids in adipose tissues directly affects the flavor of the meat. In our study, the Huangshan Black Chicken displayed higher polyunsaturated fatty acids percentage by comparing performance traits with AA broiler. Nonetheless, the precise mechanisms of Huangshan Black Chicken contributing to fatty acids composition remain unclear.

Compared with traditional cDNA microarray technologies, RNA-Seq has many advantages, such as greater dynamic range, reduced bias, lower frequency of false-positive signals, and higher reproducibility [19–20]. Moreover, the correlations between RNA-Seq and the mRNA expression level from qRT-PCR were relatively high [10,20]. Three biological replicates were used for each condition to ensure broader application in our study design, the more replicates, the greater the detection power [21].

By comparative analysis and imperative validation, we detected 274 DEGs between Huangshan black chickens with extremely high and low phenotypic values for polyunsaturated fatty acids percentage. Among them, 10 genes were identified to be located within QTL areas [22] that were affirmed to have large genetic effects on fatty acids composition, including *FADS2*, *DCN*, *FRZB*, *OGN*, *PRKAG3*, *LHFP*, *CHCHD10*, *CYTL1*, *FBLN5*, and *ADGRD1*.

Fatty Acid Desaturase 2 (*FADS2*) is one of the key limiting enzymes in the lipid metabolic pathway, which converts linoleate and alpha-linolenate into PUFAs [23]. The SNPs in the 3’ untranslated regions of the *FADS2* gene have significant genetic effects on the composition of fatty acids in gene expression activity in milk and blood [24–26]. Zhu et al [27] suggested that the SNPs of the *FADS2* gene affect the content of essential fatty acids in muscle, and played a role in the early-stage growth rate of chickens. To investigate changes in the muscle transcriptome by increased consumption of omega-6 and omega-3 fatty acids in the pig gluteus medius muscle, Ogłuszka et al [28] showed that *FADS2* may be an important gene for fatty acids metabolism. By liver transcriptome induced by a diet enriched with omega-6 and omega-3 fatty acids, Szostak et al [29] showed that *FADS2* is responsible for coding enzymes delta-6-desaturase. *FADS2* was reported to negatively regulate fat synthesis. This evidence is consistent with the results in this study. Considering the performance traits, we supposed that *FADS2* act mainly in the omega-6 metabolic pathway. Integrated analysis indicated that *FADS2* is one of the most important candidate genes for polyunsaturated fatty acids percentage in chicken.

As a small leucine-rich proteoglycan, Decorin (DCN) distributed in the extracellular matrix and reported to be associated with the cell membranes in tissues [30–31]. In the chicken, the existence of the core protein influencing two glycosaminoglycan chains has also been described [32]. DCN always acts as a ligand for the receptor tyrosine kinases including the insulin-like growth factor receptor [33] and the hepatocyte growth factor receptor [34]. Furthermore, the expression of the *DCN* gene can promote the differentiation of myoblasts, the formation of muscle fibers, and the regeneration of muscle [35]. Similarly, our study revealed that *DCN* was near to the peak positions of four QTLs for fat traits.

Frizzled motif associated with bone development (*FRZB*) is a protein-coding gene. As a member of the Wnt signaling pathway, FRZB can influence normal cellular processes through activating frizzled receptors [36]. In addition, FRZB is a competitor for the cell-surface G-protein receptor Frizzled affecting skeletal development in the embryo and fetus [37]. Bennett et al [38] have shown that *FRZB1*, *FRZB2*, and *FRZB5* are expressed in preadipocytes and mediate the repressive effects of Wnt on adipogenesis. However, Soukas et al [39] have observed that Frizzled4 is expressed in primary adipocytes but not in 3T3-L1 cells. Wang et al [40] indicated that *FRZB* expression has a positive association with fat deposition and a negative association with muscle growth and inferred that *FRZB* may be a major candidate gene for growth traits in pigs. Combined with the function of FRZB in the metabolism, our data implied that FRZB might be involved in fat metabolism through the Wnt signaling pathway.

As a regulatory subunit of the AMPK, protein kinase AMP-activated non-catalytic subunit gamma 3 (*PRKAG3*) takes part in regulating cellular energy homeostasis in a wide variety of tissues and cells [41–42]. By inactivating ACC oxidase (*ACC*) and HMG-CoA reductase *(HMGCR)*, PRKAG3 was reported to be associated with meat quality [43], which was consistent with the previous study [44]. Likewise, nucleotide variants of *PRKAG3* were able to produce significant effects on fat traits, such as final pH, meat color, and water-holding capacity in pigs [45]. Hence, *PRKAG3* was considered as a major gene affecting fat traits.

In addition, comprehensive analysis of differential expression patterns, biological functions, and QTL, revealed that six other genes, namely *OGN*, *LHFP*, *CHCHD10*, *CYTL1*, *FBLN5*, and *ADGRD1*, were also associated with fat acids composition traits to some extent. GO and IPA analysis showed that both *OGN* and *ADGRD1* are involved in accumulation of adipocyte, apoptosis, and differentiation. Osteoglycin (OGN) is involved in matrix assembly, cellular growth, and migration [46]. It is believed that OGN may be a vital humoral bone anabolic factor produced by muscle tissues [47]. Donati et al indicated that OGN regulated lipid differentiation through the Wnt/β-catenin signaling pathway [48]. Lipoma HMGIC Fusion Partner (*LHFP*) belongs to a subset of the super family of tetraspan transmembrane protein encoding genes. Mutations in the *LHFP* gene result in translocation-associated lipoma. CHCHD10 belongs to a family of mitochondrial proteins and plays a role in the mitochondrial DNA stability maintenance and mitochondrial cristae morphology [49]. CYTL1 is a cytokine-like protein specifically expressed in the bone marrow and mainly takes part in DNA repair, metabolism, and cell migration. FBLN5, a matricellular protein, plays critical roles in cell proliferation [50], vascular remodeling, and smooth muscle development [51]. Mice lacking in FBLN5 exhibit systemic elastic fiber defects [52]. As a member of the adhesion-GPCR family of receptors, ADGRD1 is involved in the control of fat and adipocyte differentiation [53]. No previous studies have linked *CHCHD10* or *CYTL1* with lipid differentiation and further study of these genes seems to be warranted.

A total of 274 genes were found to differ significantly between FAL and FAH, while some of the genes with known functions [54], e.g. *FASN*, *FABP4*, and *SCD1*, for fat acids composition and metabolism did not differ in the present study. It is likely that these genes with strong effects have been fixed by long-term genetic selection and no obvious differences have been observed between the comparison groups. It is also likely that different chicken populations were tested in previous studies.

## Conclusions

This study provided a global view of the complexity of the transcriptome of thigh muscle tissues of six Huangshan Black Chickens, and revealed 274 DEGs between Huangshan Black Chickens with extremely high and low phenotypic values of polyunsaturated fatty acids percentage. Integrated analysis of differential gene expression, QTL data and biological functions indicated that ten genes, including *FADS2*, *DCN*, *FRZB*, *OGN*, *LUM*, *LHFP*, *CHCHD10*, *CYTL1*, *FBLN5*, and *ADGRD1* represent the most promising candidates affecting meat fatty acids percentage of chicken.

## Supporting information

S1 File Performance traits of AA broilers and Huangshan Black chickens. (PDF)

S2 File Analysis of FAMEs in thigh muscle of Huangshan Black chickens. (PDF)

S3 File PCR primers for qRT-PCR validation of 10 DEGs between the two different comparison groups. (PDF)

S4 File GC-MS analysis of 37-component FAMEs standard mixture. (PDF)

S5 File The basic statistics for RNA-Seq reads generated from thigh muscle tissues with high and low fat acids percentage. (PDF)

S6 File Correlation between biological replicates within three samples. The x- and y-axis correspond to the FPKM value of each sample, respectively. The correlation coefficient (R^2^) between two individuals within each group was calculated based on the FPKM value of each individual.
(PDF)
