# Supplementary material for: RNA-Seq reveals differentially expressed genes affecting polyunsaturated fatty acids percentage in the Huangshan Black chicken population
Source: PLoS One. 2018 Apr 19;13(4):e0195132. doi: 10.1371/journal.pone.0195132 (PMC5908183; doi:10.1371/journal.pone.0195132)
Supplement: S1 File — (PDF) [file pone.0195132.s001.pdf]

## Performance traits of AA broilers and Huangshan Black chickens

| **Items** | **AA broilers (n=30, 42d)** | **Huangshan Black chickens (n=10, 120d)** |
|---|---|---|
| body weight, kg | 1.80 ±0.09 | 1.82 ±0.11 |
| percentage of eviscerated carcass, % | 78.63 ±0.47 | 71.42 ±0.40 |
| percentage of breast muscle, % | 16.55 ±0.90 | 12.59 ±1.12 |
| percentage of thigh muscle, % | 14.38 ±0.29$^{a}$ | 9.36 ±1.07$^{b}$ |
| percentage of PUFA/SFA | 0.58 ±0.06$^{A}$ | 0.75 ±0.04$^{B}$ |
| intramuscular fat, % | 2.72 ±0.37$^{A}$ | 12.68 ±1.13$^{B}$ |

**Note:** Means in the same row with different lowercase superscripts are different at P<0.05; means in the same row with different uppercase superscripts are different at P<0.01.
