# Supplementary material for: RNA-Seq reveals differentially expressed genes affecting polyunsaturated fatty acids percentage in the Huangshan Black chicken population
Source: PLoS One. 2018 Apr 19;13(4):e0195132. doi: 10.1371/journal.pone.0195132 (PMC5908183; doi:10.1371/journal.pone.0195132)
Supplement: S2 File — (PDF) [file pone.0195132.s002.pdf]

Analysis of FAMEs in thigh muscle of Huangshan Black chickens

Fatty acids (%)	Sample 1	Sample 2	Sample 3	Sample 4	Sample 5	Sample 6	Sample 7	Sample 8	Sample 9	Sample 10
C14:0	0.396	0.249	0.511	0.422	0.309	0.730	0.703	0.289	0.305	0.425
C16:0	18.949	19.882	20.507	20.845	19.308	21.623	21.484	20.275	18.100	19.289
C18:0	19.298	22.493	17.606	19.756	19.137	16.110	14.116	20.570	22.681	18.987
C20:0	0.335	0.350	0.277	0.234	0.355	0.207	0.164	0.225	0.254	0.256
C14:1n-5	0.124	0.041	0.168	0.055	0.069	0.154	0.186	0.072	0.089	0.112
C16:1n-7	1.668	1.184	3.304	1.596	1.231	3.902	5.849	1.206	1.498	2.015
C18:1n-9	23.548	19.422	25.504	22.012	22.812	27.516	26.644	20.796	21.500	24.478
C18:1n-7	2.439	3.464	2.747	2.494	2.924	2.840	3.115	3.452	3.230	3.491
C20:1n-9	0.267	0.274	0.319	0.211	0.339	0.260	0.193	0.266	0.241	0.262
C18:2n-6	18.674	14.997	15.786	16.461	18.328	15.110	15.729	18.329	17.597	16.685
C18:3n-6	0.207	0.197	0.159	0.227	0.124	0.275	0.208	0.119	0.149	0.178
C18:3n-3	0.253	0.193	0.245	0.215	0.210	0.248	0.303	0.143	0.226	0.307
C20:2n-6	0.336	0.359	0.305	0.268	0.113	0.150	0.259	0.260	0.225	0.309
C20:3n-3	0.015	0.501	0.241	0.134	0.213	0.017	0.246	0.568	0.744	0.303
C20:4n-6	9.679	11.156	8.838	10.426	9.658	7.814	6.530	9.521	9.803	10.497
C20:5n-3	0.061	0.137	0.155	0.032	0.098	0.000	0.515	0.187	0.231	0.049
C22:6n-3	2.935	4.073	2.665	4.106	3.832	2.294	2.389	3.052	2.390	1.606
SFA	38.978	42.975	38.901	41.257	39.109	38.670	36.466	41.359	41.340	38.957
PUFA	32.160	31.614	28.395	31.601	32.577	25.909	26.178	32.178	31.364	29.934
PUFA/SFA	**0.825**	0.735	**0.629**	0.766	**0.832**	**0.670**	**0.717**	**0.778**	0.758	0.768

Note: SFA = C14:0 + C16:0 + C18:0 + C20:0; MUFA = C14:1 + C16:1 + C18:1 + C18:1 + C20:1; PUFA = C18:2 + C18:3 + C18:3 + C20:2 + C20:3 + C20:4 + C20:5 + C22:6; USFA = MUFA + PUFA. Of these, six samples correspond respectively with (sample1-FAH1, sample5-FAH2, sample8-FAH3; sample3-FAL1, sample6-FAL2, sample7-FAL3).
