# Supplementary material for: RNA-Seq reveals differentially expressed genes affecting polyunsaturated fatty acids percentage in the Huangshan Black chicken population
Source: PLoS One. 2018 Apr 19;13(4):e0195132. doi: 10.1371/journal.pone.0195132 (PMC5908183; doi:10.1371/journal.pone.0195132)
Supplement: S3 File — (PDF) [file pone.0195132.s003.pdf]

PCR primers for qRT-PCR validation of 10 DEGs between the two different comparison groups

Gene	Primer sequences (5'to3')	Length（bp）
FADS2-F	TGAGGAAGACAGCAGAGGACAT	152
FADS2-R	GCAGGCAAGGATTAGAGTTGTGA	
ABI3BP-F	GGTTCTACACGGTTCGCTACAG	152
ABI3BP -R	GCTCCAGACGCTATCCTCCAT	
DCN-F	CGCATCGCAGACACCAACA	112
DCN-R	CAGACAGACCTTCCGCATCAAT	
LUM-F	GACTGCCTCACTCCTTACTCAT	153
LUM-R	TATTGAAGACATTGCCTGGTATTCC	
FRZB-F	CCAGAAGACCTATCTACGCAACAA	260
FRZB-R	AGCCTTCCACCAAGAGTAACCT	
OGN-F	TGAAGCCAGCACCACCTATACA	235
OGN-R	CACACGCAGAGCAGACAAGTAG	
CA10-F	AAGGCAGTCACCCGTCAATA	237
CA10-R	GATCCCTGGCTGTCTTCACT	
EDA2R-F	GGAGACCGAGTACCTGGATG	213
EDA2R-R	CAGTTGGACTTCTGGATGCG	
ZIC4-F	ACCATCACCACCACCATCAT	160
ZIC4-R	ATTAGCCAGTCCCTACCTGC	
FBLN1-F	GGTCAAGAAGGCACGGATGT	378
FBLN1-R	ACCAGTCTCACATTCGTCAATATCT	
18s rRNA -F	TAGATAACCTCGAGCCGATCGCA	312
18s rRNA -R	GACTTGCCCTCCAATGGATCCTC	
β-actin -F	CTGTGCCCATCTATGAAGGCTA	139
β-actin -R	ATTTCTCTCTCGGCTGTGGTG	
GAPDH-F	AACTTTGGCATTGTGGAGGG	130
GAPDH-R	ACGCTGGGATGATGTTCTGG	
