# Supplementary figures and images for: RNA-Seq reveals differentially expressed genes affecting polyunsaturated fatty acids percentage in the Huangshan Black chicken population
Source: PLoS One. 2018 Apr 19;13(4):e0195132. doi: 10.1371/journal.pone.0195132 (PMC5908183; doi:10.1371/journal.pone.0195132)

GC-MS analysis of 37-component FAMES standard mixture

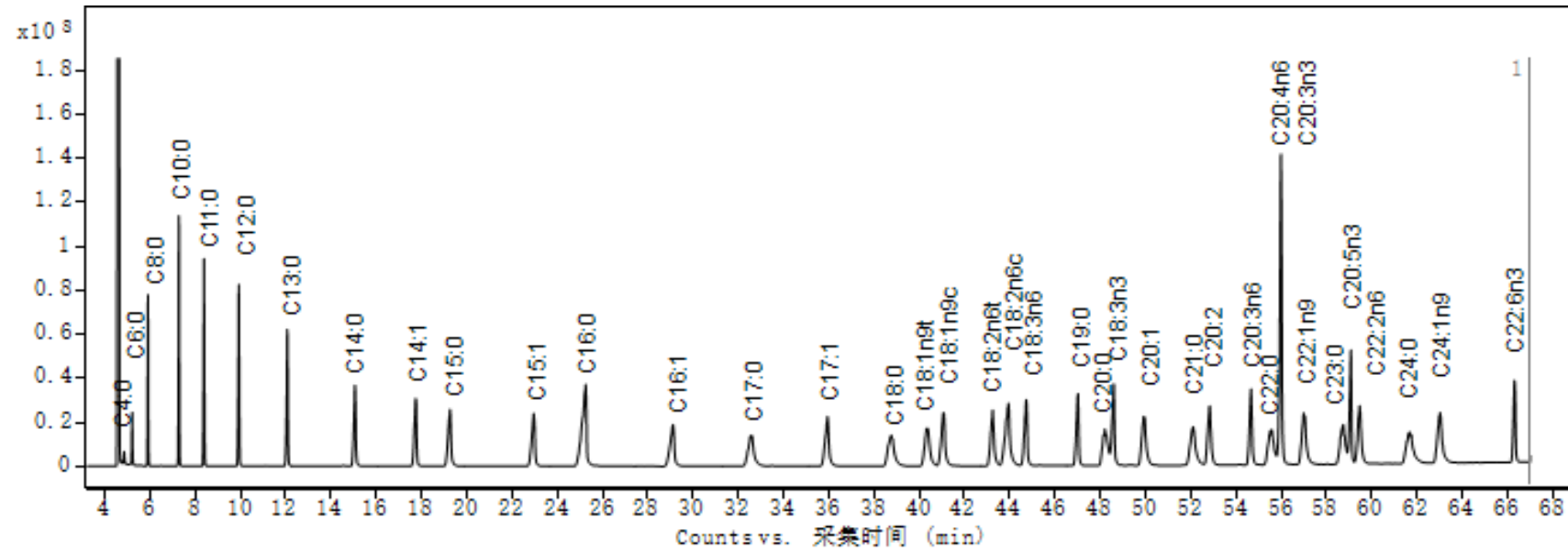

Supplement: S4 File — (PDF) [file pone.0195132.s004.pdf]

Correlation between biological replicates within three samples

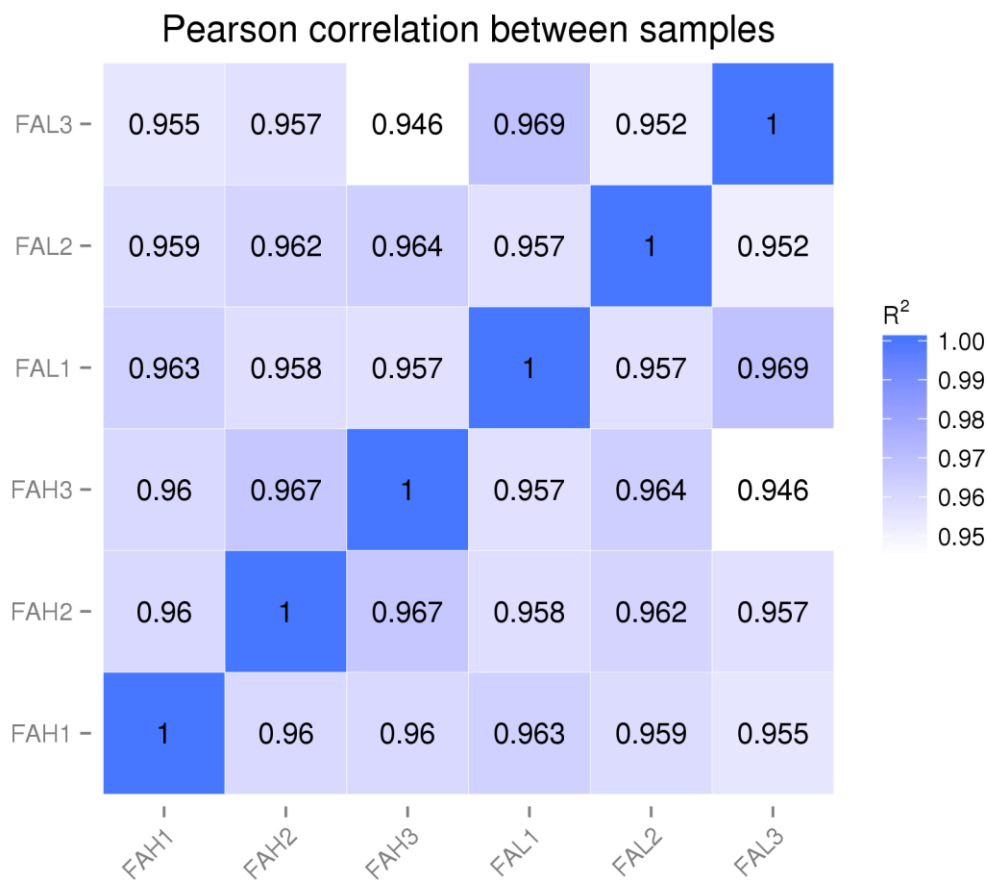

Supplement: S6 File — The x- and y-axis correspond to the FPKM value of each sample, respectively. The correlation coefficient (R2) between two individuals within each group was calculated based on the FPKM value of each individual. (PDF) [file pone.0195132.s006.pdf]
