# Supplementary material for: RNA-Seq reveals differentially expressed genes affecting polyunsaturated fatty acids percentage in the Huangshan Black chicken population
Source: PLoS One. 2018 Apr 19;13(4):e0195132. doi: 10.1371/journal.pone.0195132 (PMC5908183; doi:10.1371/journal.pone.0195132)
Supplement: S5 File — (PDF) [file pone.0195132.s005.pdf]

The basic statistics for RNA-seq reads generated from thigh muscle tissues with high fat acid percentage and low fat acid percentage

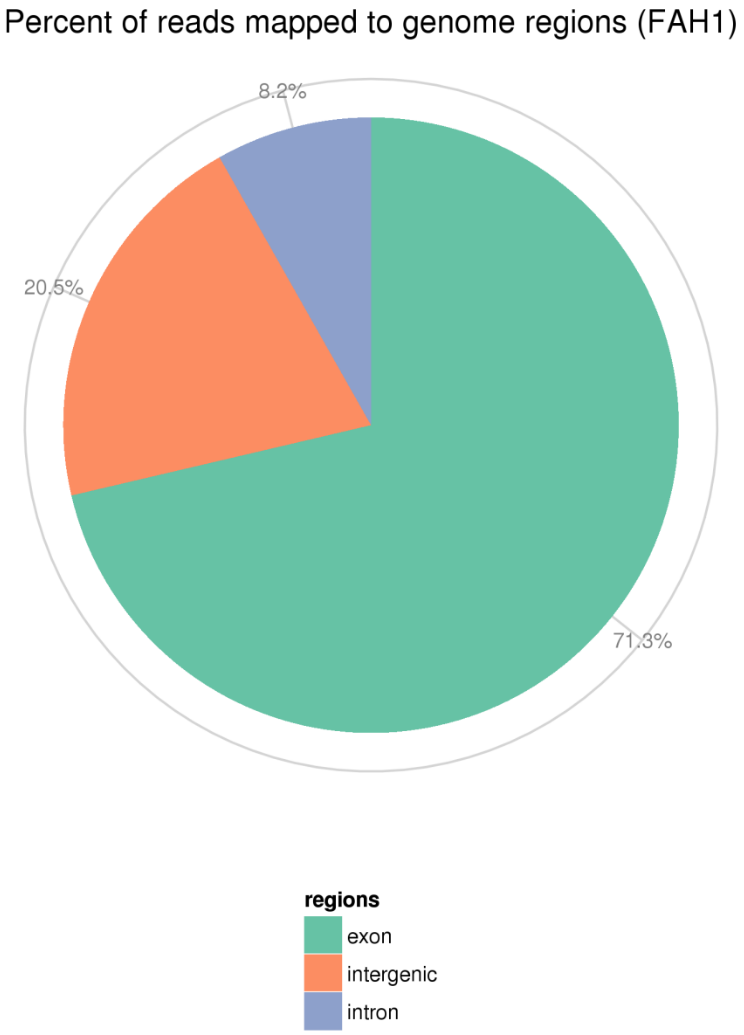

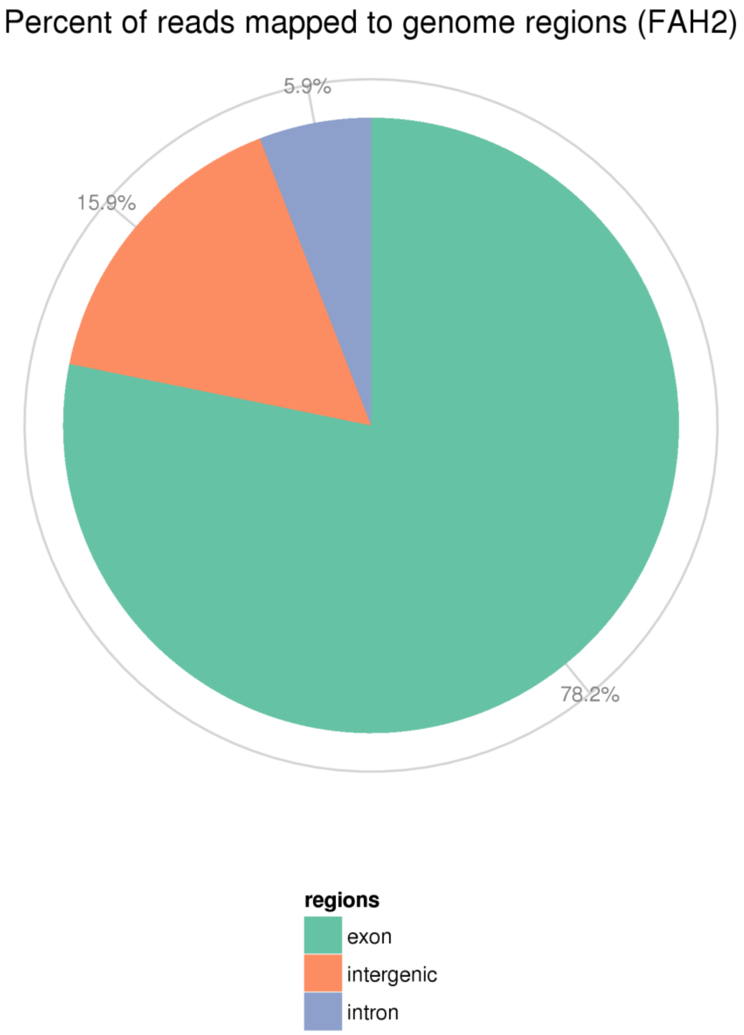
Percent of reads mapped to genome regions (FAH2)
5.9%
15.9%
78.2%
regions
exon
intergenic
intron

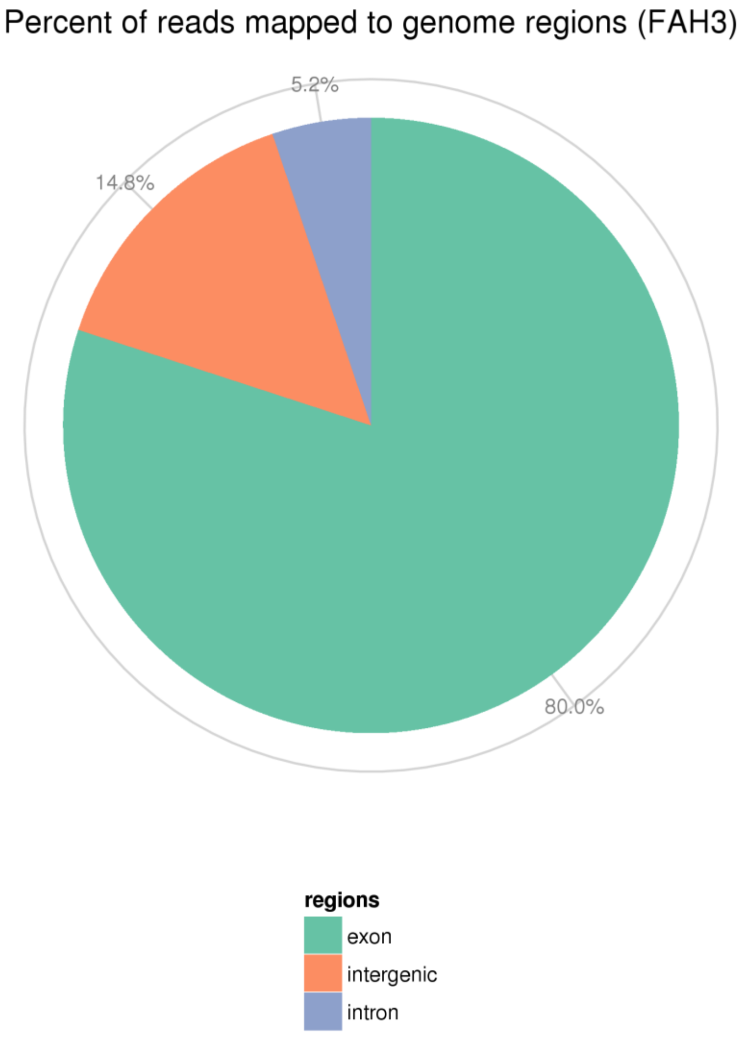

Percent of reads mapped to genome regions (FAH3)
5.2%
14.8%
80.0%
regions
exon
intergenic
intron

Percent of reads mapped to genome regions (FAL1)

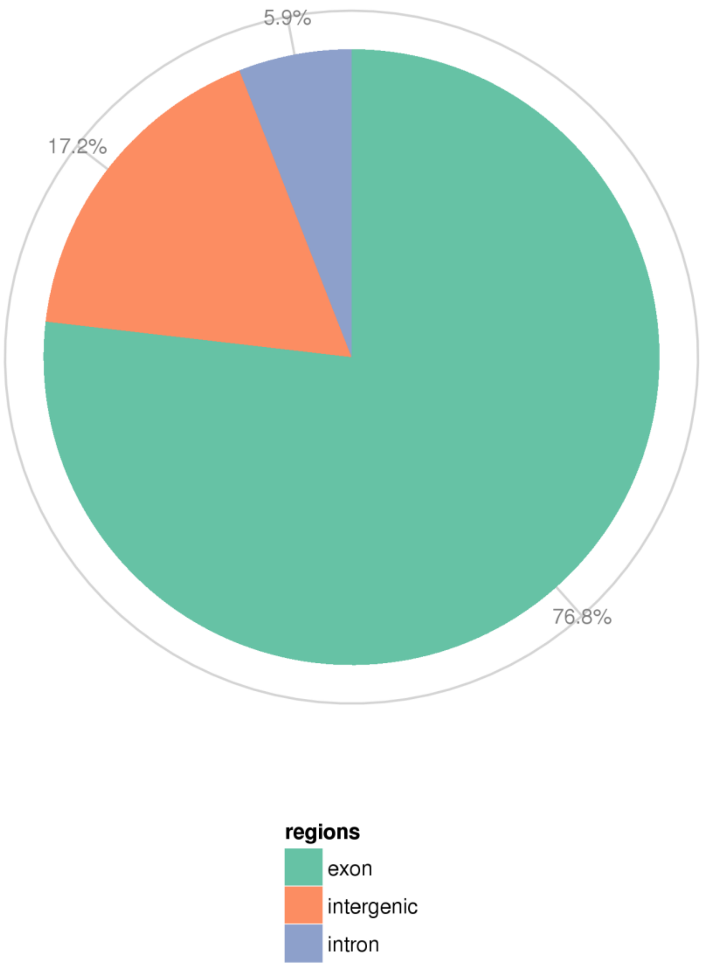

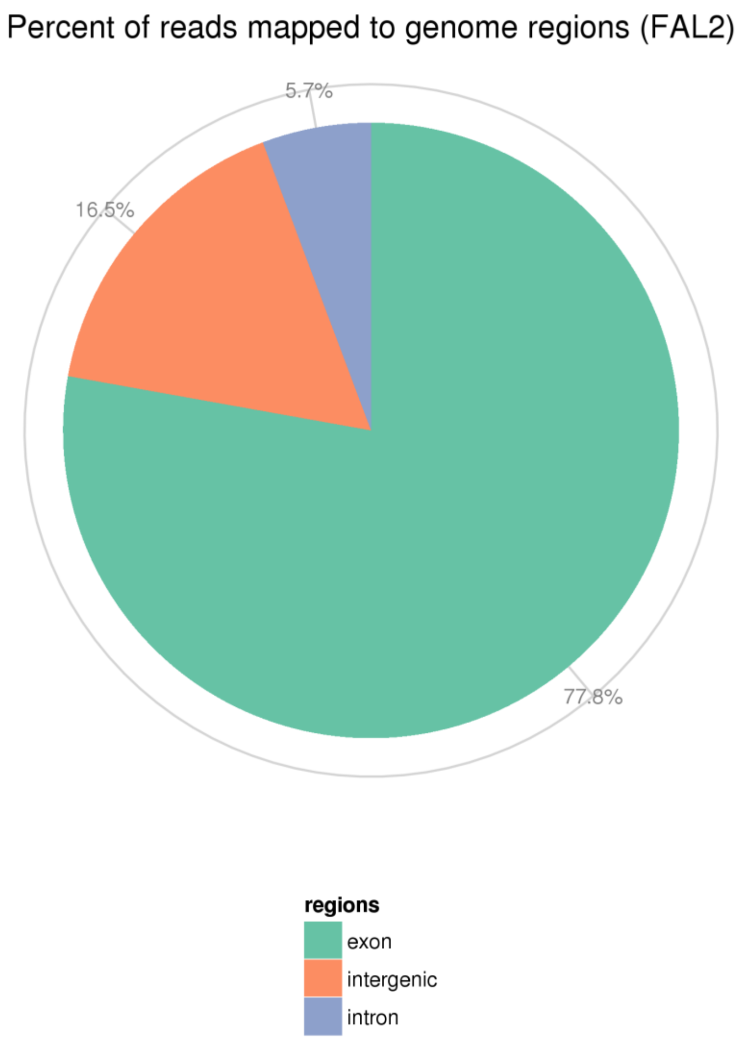
Percent of reads mapped to genome regions (FAL2)
5.7%
16.5%
77.8%
regions
exon
intergenic
intron

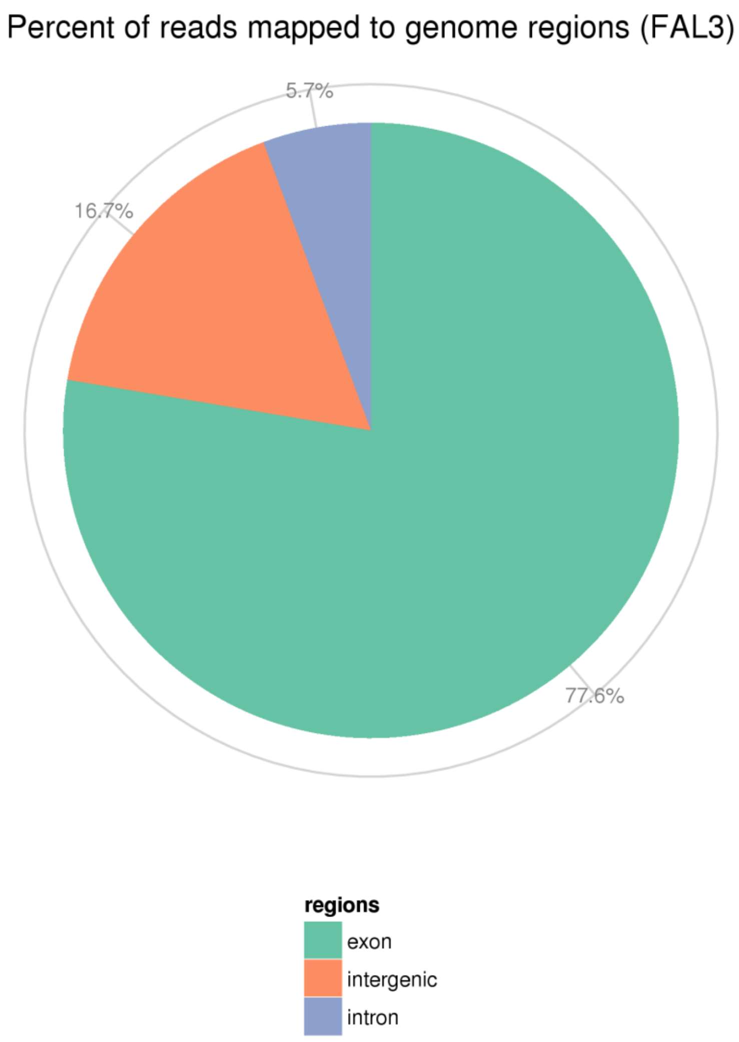
Percent of reads mapped to genome regions (FAL3)
5.7%
16.7%
77.6%
regions
exon
intergenic
intron
